# Dextran-coated iron oxide nanoparticle-induced nanotoxicity in neuron cultures

**DOI:** 10.1038/s41598-020-67724-w

**Published:** 2020-07-08

**Authors:** Ryan P. Badman, Shanna L. Moore, Jessica L. Killian, Tuancheng Feng, Thomas A. Cleland, Fenghua Hu, Michelle D. Wang

**Affiliations:** 1 Department of Physics and LASSP, Cornell University, Ithaca, NY 14853 USA; 2 Howard Hughes Medical Institute, Cornell University, Ithaca, NY 14853 USA; 3 Department of Molecular Biology and Genetics, Cornell University, Ithaca, NY 14853 USA; 4 Weill Institute for Cell and Molecular Biology, Cornell University, Ithaca, NY 14853 USA; 5 Department of Psychology, Cornell University, Ithaca, NY 14853 USA; 6 Present Address: Center for Brain Science, RIKEN, Saitama, 351-0198 Japan; 7 Present Address: Quantum Biosystems, Menlo Park, CA 94025 USA

**Keywords:** Neuroscience, Physiology

## Abstract

Recent technological advances have introduced diverse engineered nanoparticles (ENPs) into our air, water, medicine, cosmetics, clothing, and food. However, the health and environmental effects of these increasingly common ENPs are still not well understood. In particular, potential neurological effects are one of the most poorly understood areas of nanoparticle toxicology (nanotoxicology), in that low-to-moderate neurotoxicity can be subtle and difficult to measure. Culturing primary neuron explants on planar microelectrode arrays (MEAs) has emerged as one of the most promising in vitro techniques with which to study neuro-nanotoxicology, as MEAs enable the fluorescent tracking of nanoparticles together with neuronal electrical activity recording at the submillisecond time scale, enabling the resolution of individual action potentials. Here we examine the dose-dependent neurotoxicity of dextran-coated iron oxide nanoparticles (dIONPs), a common type of functionalized ENP used in biomedical applications, on cultured primary neurons harvested from postnatal day 0–1 mouse brains. A range of dIONP concentrations (5–40 µg/ml) were added to neuron cultures, and cells were plated either onto well plates for live cell, fluorescent reactive oxidative species (ROS) and viability observations, or onto planar microelectrode arrays (MEAs) for electrophysiological measurements. Below 10 µg/ml, there were no dose-dependent cellular ROS increases or effects in MEA bursting behavior at sub-lethal dosages. However, above 20 µg/ml, cell death was obvious and widespread. Our findings demonstrate a significant dIONP toxicity in cultured neurons at concentrations previously reported to be safe for stem cells and other non-neuronal cell types.

## Introduction

Industrialization and the recent surge in nanotechnology commercialization has greatly increased human exposure to engineered nanoparticles (ENPs)^1–3^, which are defined as man-made particles < 100 nm in all dimensions. This recent and sudden ubiquity of ENPs is a drastic shift in the human environment, and concerns have been mounting that regulatory agencies have not been adequately vetting the health impacts of products and industrial processes utilizing ENPs before they are introduced to the public^4,5^.

Unlike larger particles, nanoparticles can rapidly enter the body through inhalation, ingestion, or dermal absorption, and then quickly travel to cells and to subcellular structures in organs throughout the body^6^. A notable exception is the mammalian central nervous system (CNS), which experiences considerable protection against invasion by most nano- or microparticles^7^, primarily due to the blood–brain-barrier (BBB). However, significant CNS exposure to ENPs can occur both advertently, through designing functionalized ENPs that can penetrate the CNS for biomedical applications^7^, and inadvertently through concentrated pollution where ENPs enter the bloodstream, potentially damaging the BBB, reaching the CNS, or overwhelming the CNS’s olfactory bulb port of entry during inhalation^8,9^.

Functionalized iron oxide nanoparticles (IONPs) are ENPs that may soon be commonly employed for applications in the CNS^10–17^. Although functionalized IONPs, especially dextran-coated IONPs (dIONPs) or IONPs coated with similar carbohydrate coatings, have been reported to have very low toxicity in the liver in a handful of clinical trials^16^, toxicity results from these studies may not be broadly applicable to other cell types because liver cells have among the highest iron tolerances in the mammalian body^18^. There are several limited in vitro and in vivo results demonstrating that there may be detrimental long-term effects of dIONPs in organs beyond the liver^15,17,19,20^, and iron accumulation is increasingly implicated in neurodegenerative diseases^17,21^ Additionally, because IONPs have almost molecular size, they have a high surface-to-volume ratio. Thus their reactivity is much higher than larger particles and their interactions with subcellular structures can change dramatically with just slight alterations in ENP shape or surface properties^22,23^. While progress has been made in optimizing low-toxicity IONP coatings for applications that do not require cell internalization, such as drug delivery^24,25^ or vascular imaging^26^, challenges remain in synthesizing safe coatings on IONPs for applications that require neuronal cell uptake for long-term labeling^15^.

Since many proposed biomedical applications using dIONPs involve mammalian CNS neurons^13,15^, it is important to establish toxicity thresholds, kinetics, and toxicity mechanisms of dIONPs using in vitro neuron culture^23^. In nanotoxicology in particular, the majority of studies utilize accessible in vitro systems due to the current lack of federal regulatory standards for implementing in vivo studies^4,23^. In vitro toxicology is most physiologically relevant when primary cells are used instead of immortalized cell lines^27^. To assess the safety of exposing the mammalian CNS to dIONPs, we explored their effects on mature, primary mammalian (murine) neuron health, in terms of both cell viability and electrophysiological activity measured with planar microelectrode arrays (MEAs)^28^. Murine cultures become electrically mature quickly (within 2–3 weeks) as compared to human induced pluripotent stem cell-derived neuron cultures which take many months^29^.

Therefore, the safety of dIONPs in the mammalian brain is not well understood, especially for cell-labeling and cell uptake applications^15^. Whereas uncoated IONPs have been studied on MEAs (albeit interpretation of toxicological implications limited by large IONP size variation)^30^, there has, to date, been no investigation of the effects of biomedically-coated iron oxide ENPs on the survival and electrical activity of fully differentiated primary neurons. In this work, we chose Molday ION Rhodamine B (MIRB) nanoparticles^31^, a commercially available dual magnetic/fluorescence dIONP. MIRB nanoparticles have an 8 nm iron oxide (magnetite) core, a size range optimal for magnetic resonance imaging (MRI) applications^32^ and cellular uptake (generally, particles < 100 nm are more readily taken up by cells)^33^. The commercial availability of MIRB nanoparticles also enables direct comparison to other MIRB studies. This is advantageous since researchers have raised concerns that use of non-standardized ENPs, such as in-house prepared particles, hinders the comparison of nanotoxicology studies from different research laboratories^4,23,34^. Here, we provide evidence that dIONPs are toxic to neurons at doses reported to be safe for other cell types in vitro, indicating that caution should be used when employing ENPs in the brain.

## Results

### MIRB intracellular distribution and uptake kinetics

**Figure 1:**
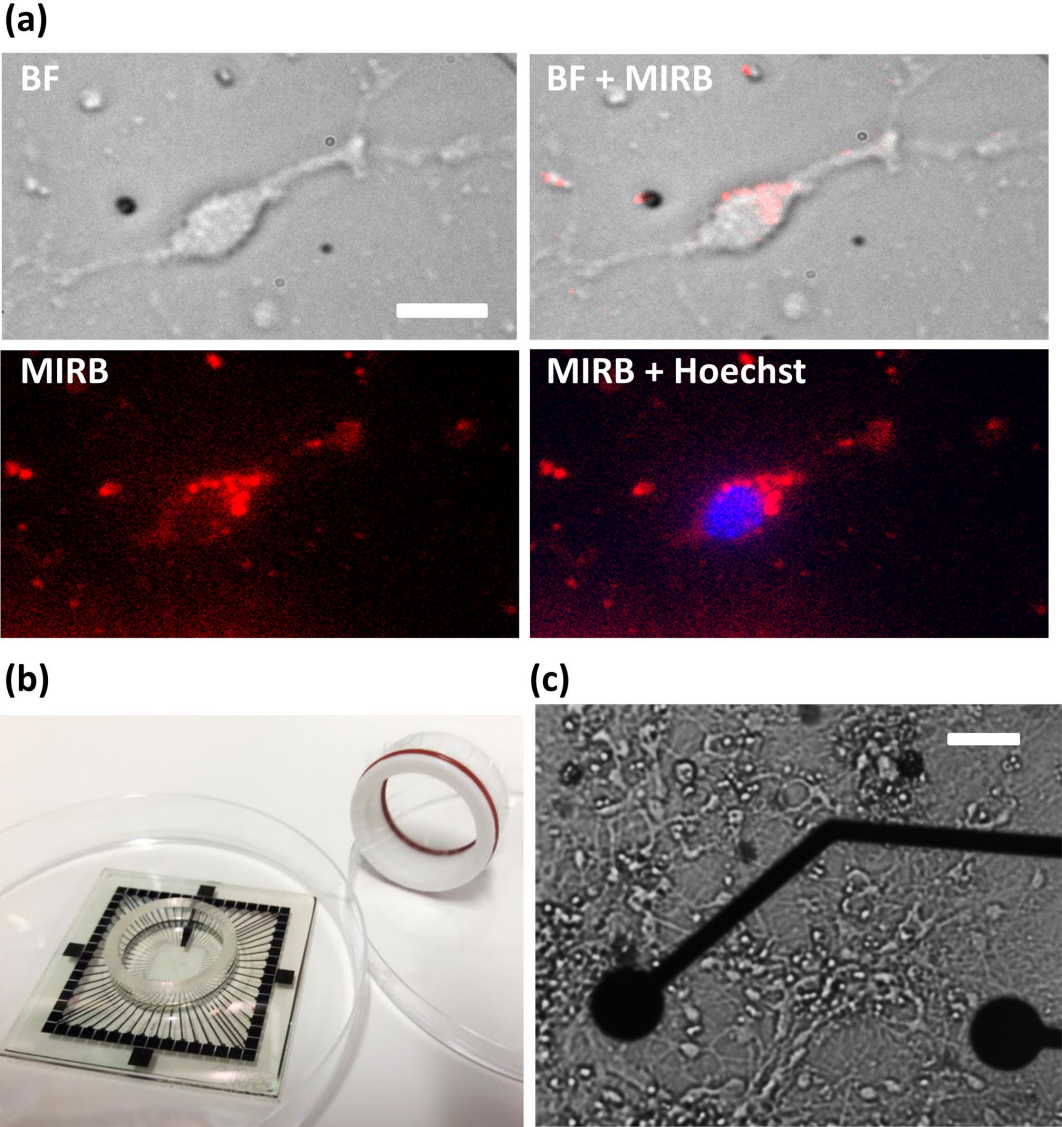
MIRB uptake by murine neurons in an MEA culture chamber. (**a**) Localization of MIRB nanoparticles in neurons. DIV19 neurons were incubated for 2 h with 10 µg/ml MIRB nanoparticles before the particles were washed out. Both bright field and fluorescence images were acquired at 24 h subsequent to the start of this incubation (DIV20). The fluorescence image of MIRB nanoparticles is shown in red; the fluorescence image of Hoechst 33342 stain is shown in blue. Scale bar is 20 µm. (**b**) A photograph of a microelectrode array (MEA) and specialized MEA lid used in this study. The MEA is placed inside a 100 mm culture dish for size reference. (**c**) A bright field image of a typical neuron culture (DIV19) plated in a MEA chamber. Scale bar is 30 µm.

**Figure 2:**
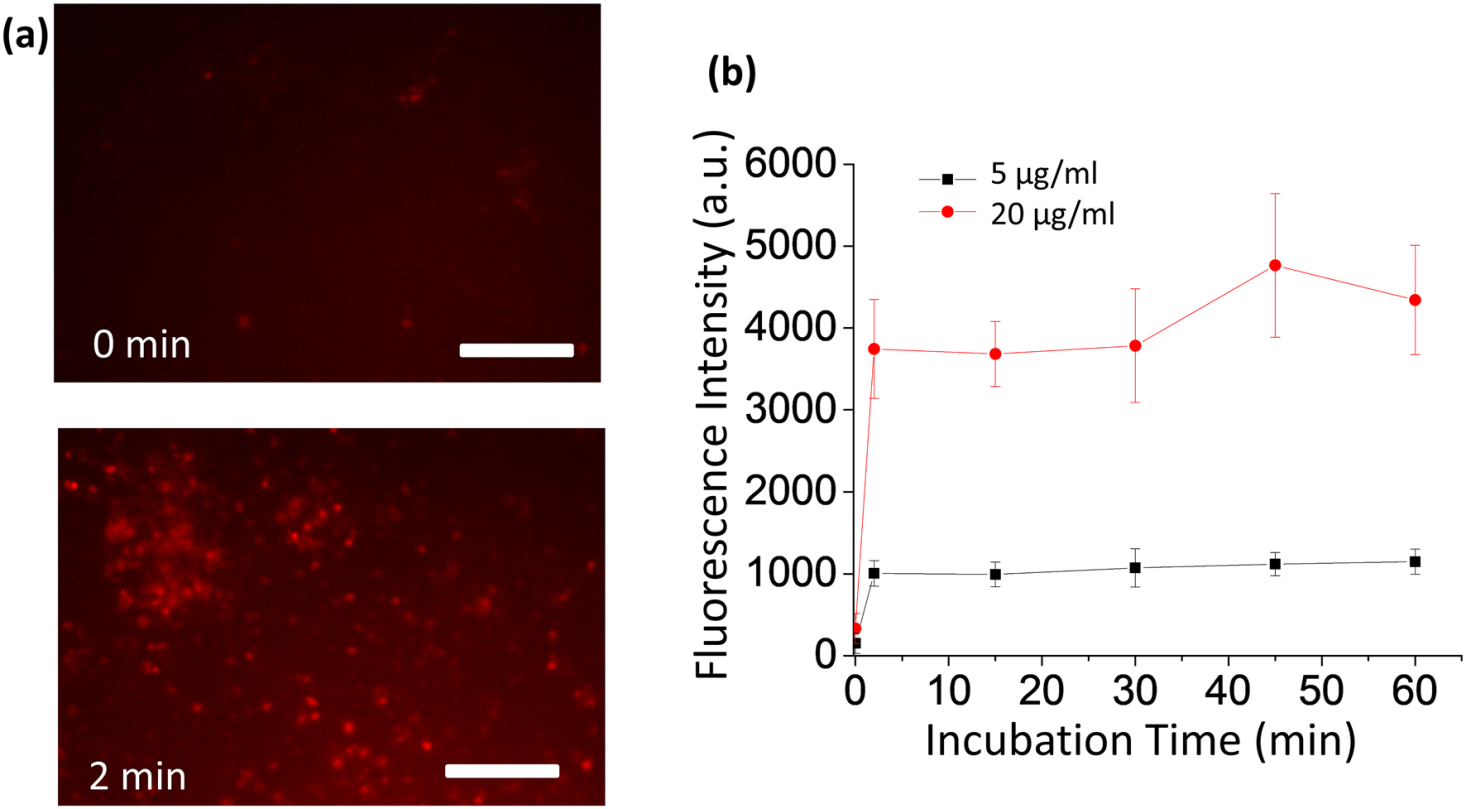
MIRB Nanoparticle Localization to Neural Cells. (**a**) Fluorescent images of clusters of neurons before exposure to MIRB nanoparticles (0 min, top insert) and after 2 min of MIRB exposure followed by the removal of free particles (2 min, bottom insert). Scale bar is 100 µm. (**b**) Kinetics of MIRB association with neurons measured by fluorescent images of MIRB nanoparticles. Neurons were incubated with MIRB nanoparticles at either 5 or 20 µg/ml for the specified duration before removal of free particles. Each error bar denotes the standard deviation of the mean values of all ROIs at a given time point and dose.

To guide the interpretation of toxicity results in primary neuron cultures (Fig. 1), we first verified that fluorescent MIRB nanoparticles interacted with neurons by observing MIRB cellular uptake and co-localization with neurons. All MIRB dosages presented observable fluorescent signals that were strongly spatially correlated with cultured neurons (Fig. 1a, Supplementary Fig. 1). Presumably due to electrostatic interactions between the positively charged MIRB nanoparticles and the negatively charged neuronal cell membranes^35,36^, particle association with the neurons was extremely rapid, with the fluorescent signal plateauing in less than two minutes (Fig. 2). After 24 h, fluorescent signal was strong in cytoplasmic regions^5,37^, but absent from nuclear regions (Fig. 1a, Supplementary Fig. 1). This observation strongly implies successful MIRB particle uptake and neuronal internalization, which is expected given previous literature findings that surface-charged dextran iron oxide nanoparticles are taken up by charge-mediated endocytosis^36,38^.

The small microglial cells in culture were also observed to take up MIRB particles, as assessed by bright field microscopy overlaid with MIRB fluorescent data, but we did not further study microglial response to MIRB nanoparticles. Previous work, however, has shown microglial activation and inflammation response to MIRB nanoparticle uptake after particle leakage from nearby MIRB-tagged grafted neural stem cells, or their derivatives, in the murine brain^15^.

In summary, we conclude that (1) initial MIRB nanoparticles’ association with neural cells occurs rapidly (< 2 min), likely at the cell membrane, and (2) subsequent neuronal internalization of MIRB nanoparticles significantly plateaus after 24 h.

## ROS imaging and neuron viability

Reactive oxidative species (ROS) studies were chosen as neurons are particularly sensitive to the subcellular damage that can result from the oxidative stress and inflammation known to be triggered by excess ROS within a cell. IONPs have the potential to generate significant ROS response if the particle coating is being digested by the cell, exposing bare iron oxide^38,39^.

**Figure 3:**
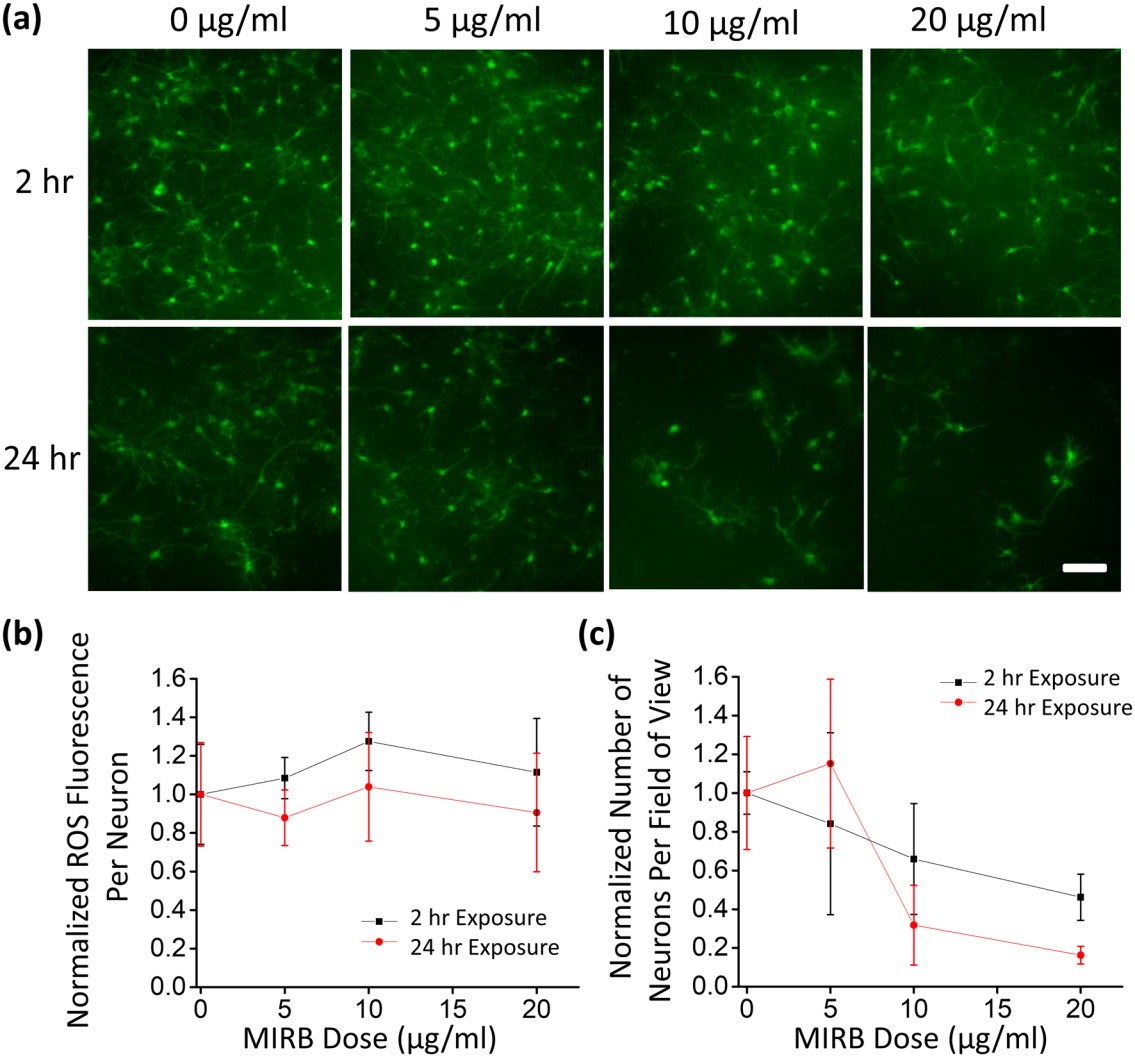
ROS staining of neurons for ROS and viability measurements. (**a**) DIV19 neurons were incubated with MIRB nanoparticles for 2 h (top row) or 24 h (bottom row) at dosages of 0 µg/ml (control), 5 µg/ml, 10 µg/ml or 20 µg/ml. Live neurons were fluorescently observed with a fluorometric intracellular ROS kit (green) particularly sensitive to superoxide and hydroxyl radicals. Scale bar is 100 µm. (**b**) Plot of the ROS fluorescence intensity per neuron normalized against that of the control. (**c**) Plot of the number of fluorescent neurons per field of view normalized against that of the control. Each error bar denotes the standard deviation of the mean values of the replicate wells at a given time point and dose.

All cultured neurons controls without MIRB addition showed a similar and clearly observable native ROS signal, and all MIRB-exposed wells had mean ROS fluorescence levels within ~ 20% of the controls’ mean ROS signal (Fig. 3a, b). An observable baseline ROS signal in controls is expected, given that neurons are among the most metabolically active cells in the body and thus naturally produce large amounts of ROS as metabolic byproducts^40^. In contrast, the smaller glial cells in the cultures did not produce a readily observable baseline ROS signal. The ROS-dependent fluorescent signal in individual neurons did not change significantly as a function of MIRB dosage (Fig. 3b).

Fewer ROS neurons per field of view were observed as the MIRB dosage increased, which we interpreted as neuronal death, i.e., the absence of ROS signal resulted from the lack of metabolic activity. Specifically, whereas 0 µg/ml (control) and 5 µg/ml dosages showed comparable numbers of metabolically active neurons per field of view after both 2, 24, and 48 h incubation times, higher dosages presented clear reductions in the numbers of surviving neurons, particularly in the samples incubated with MIRB nanoparticles for 24 h or 48 h (Fig. 3a,c; Supplementary Fig. 2). It is possible that this is caused by the rapid sheathing of neuron cell membranes by the MIRB nanoparticles prior to internalization, leading to a disruption of neuronal nutrient intake or other critical cell membrane functions^35^, with the lethal dosage depending to some degree on exposure time (Fig. 3, Supplementary Fig. 2).

The lack of ROS increase subsequent to MIRB incubation (Fig. 3a, b) is consistent with previous work examining comparably sized iron oxide core particles, which showed negligible increase in ROS generation after exposure to 5–30 nm core dIONPs, but significant increase in ROS generation with bare IONPs that leach iron more readily^38^. The negligible effects of MIRB nanoparticles on ROS generation seen in this work suggest that the particle coatings remained intact during and after cell uptake, thereby preventing iron leaching and subsequent ROS formation from the Fenton and/or Haber–Weiss reactions^41^.

The cell viability trends observed in the ROS assay were further validated by use of a kit that uses a live cell, green fluorescent, cell-permeable viability stain (Calcein AM)^42^. The Calcein AM fluoresces only after cytosolic esterase enzymatic interactions within healthy, metabolically active cells and has low or no signal within dead cells (Supplementary Fig. 3). Additionally, the dye is an accepted indicator of intact cell membranes^42^. In agreement with results observed with the ROS assay, obvious gross cell toxicity was observed at the 10 and 20 µg/ml doses after 24 h nanoparticle incubation.

### Electrophysiology

We next studied whether increasing MIRB dosage affects electrical communication between neurons. Specifically, we examined the electrical activities of neuron cultures plated on MEAs by systematically characterizing commonly studied MEA parameters: the number of active electrodes, spike rate, burst rate, burst duration, and the number of spikes per burst^30,43,44^.

**Figure 4:**
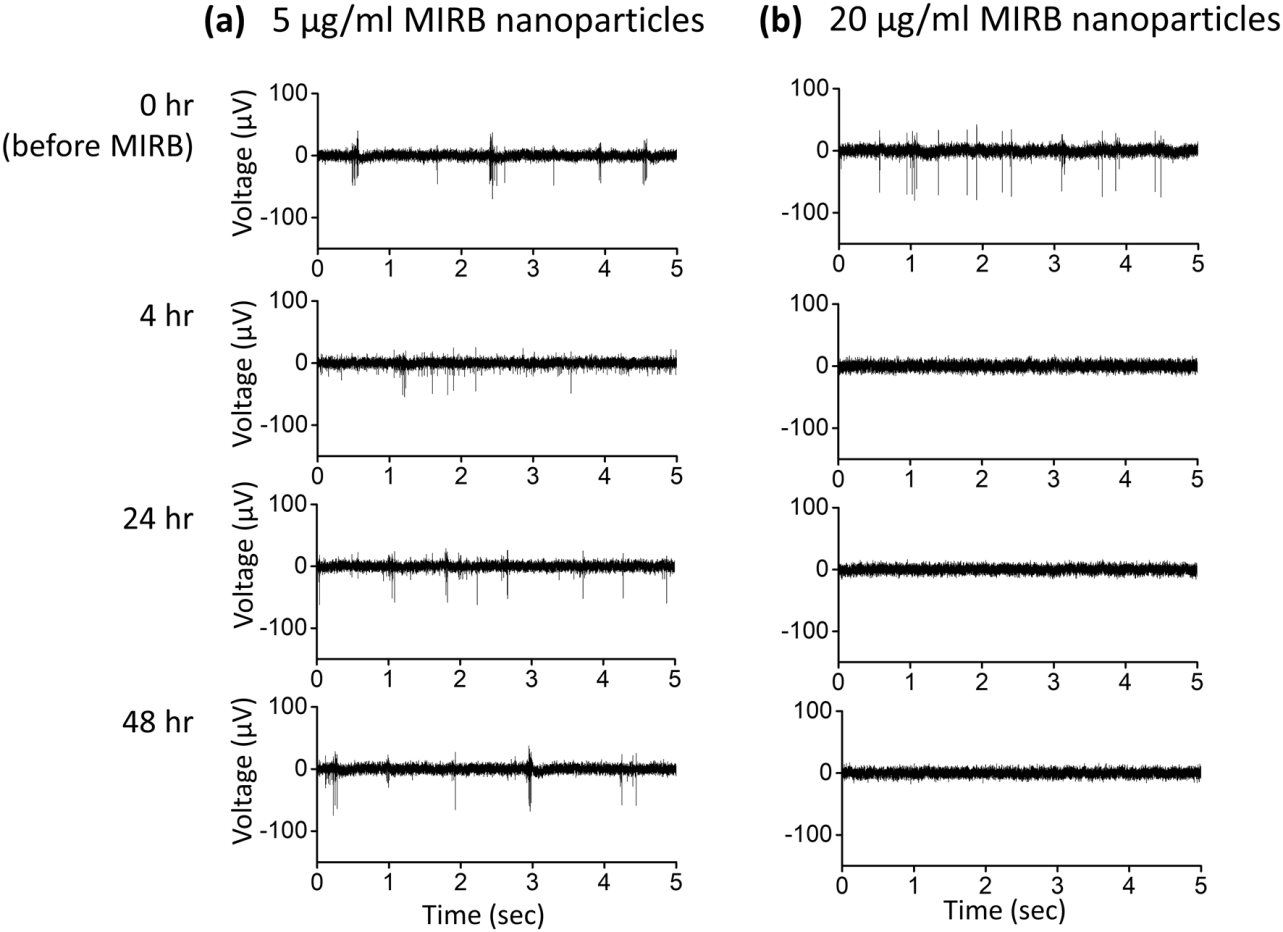
Example electrophysiological time course for two active electrodes at different doses. Example raw spike traces from an active electrode versus time after exposure to (**a**) 5 µg/ml MIRB nanoparticles and (**b**) 20 µg/ml MIRB nanoparticles. In both cases, MIRB nanoparticles were incubated for 2 h before removal (removal being 2 h before the 4 h time point).

**Figure 5:**
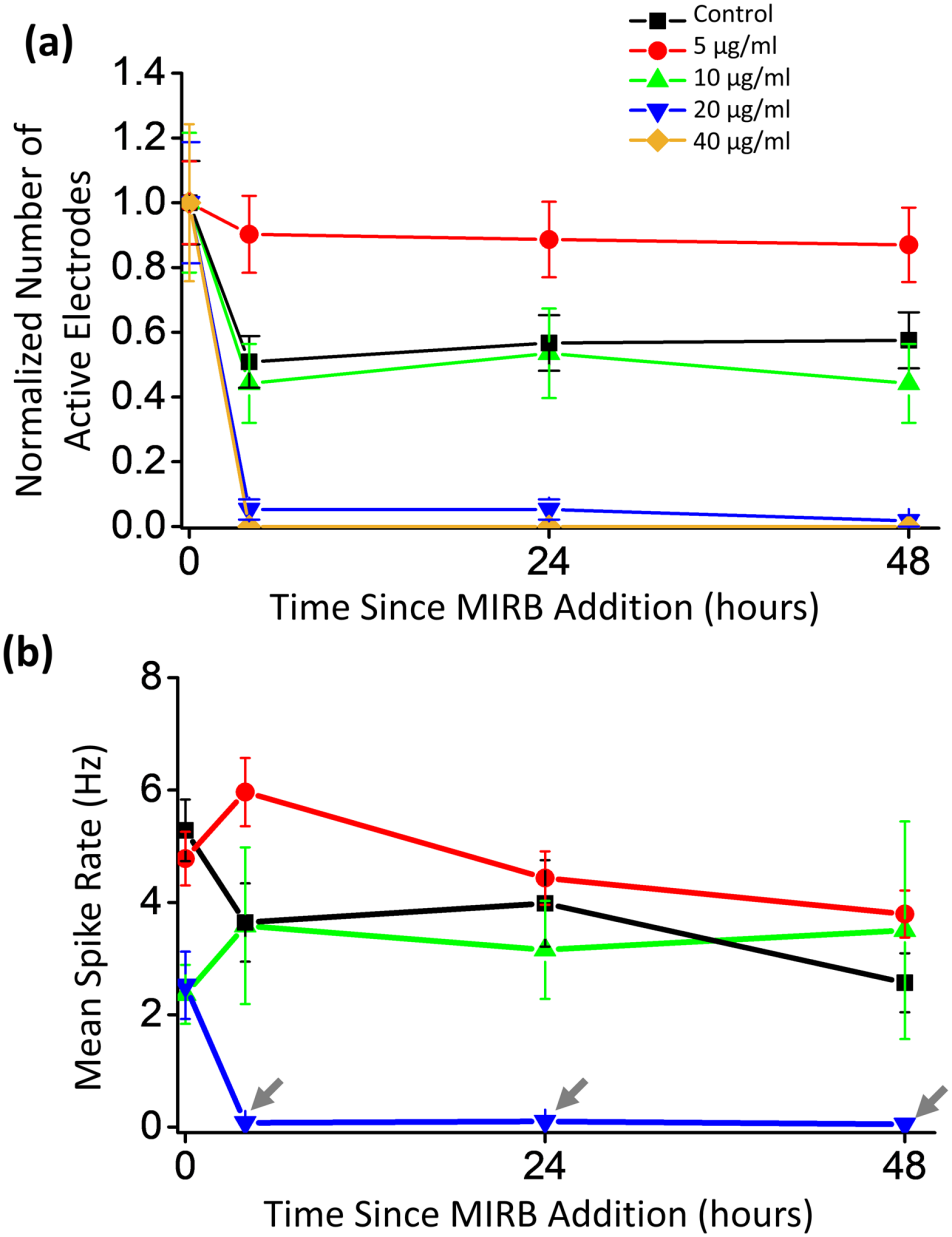
Dose-dependent active electrode count and mean spike rate versus time for MEA neuron cultures. (**a**) Plot of total number of active electrodes versus time, normalized to the total number of active electrodes originally spiking at a given MIRB dose at 0 h (pretreatment). For each dose, MIRB nanoparticles were incubated for 2 h before removal. At the dose of 40 µg/ml, no active electrodes were detected at 4 h or later and those MEA data were not further analyzed. We found that prior to incubation with 0 (control), 5, 10, 20, or 40 µg/ml MIRB nanoparticles, there were 30, 41, 15, 19, and 17 active electrodes (> 0.03 Hz) per MEA on average (out of 60 possible electrodes), respectively. Error bars are Poisson counting errors. (**b**) Mean spike rates of active electrodes versus time. Error bars denote standard error of the mean.

**Figure 6:**
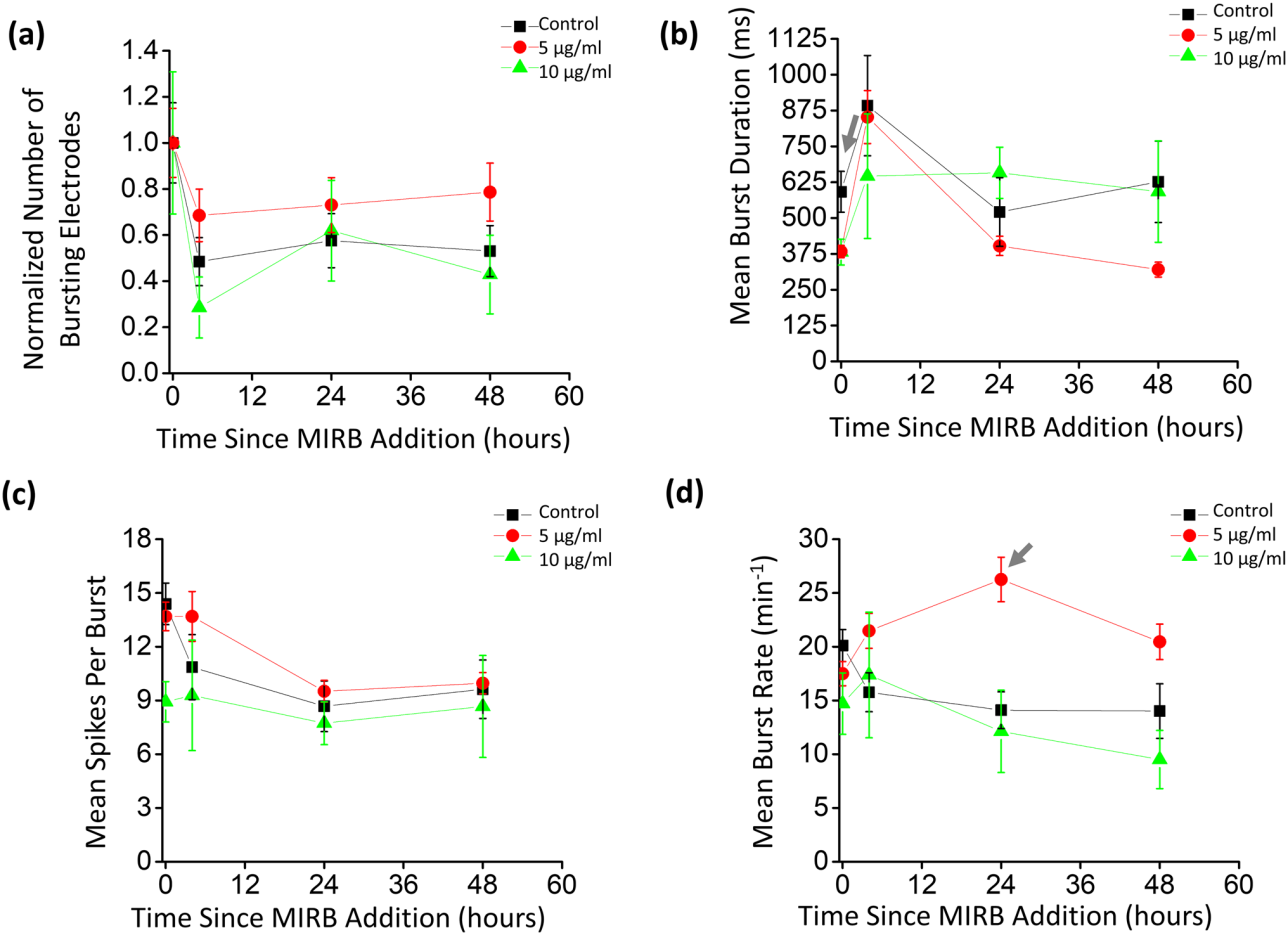
Dose-dependent bursting properties of MEA neuron cultures over time. MEA electrode burst analyses were performed for non-lethal MIRB dosages of 0 µg/ml, 5 µg/ml, and 10 µg/ml, at time points of 0 h (pretreatment; ~ 1 h prior to MIRB addition), and 4, 24, and 48 h subsequent to MIRB addition. (**a**) Total number of bursting electrodes, normalized to the total number of bursting electrodes at 0 h (pretreatment). For this burst analyses performed with 0 (control), 5, and 10 µg/ml MIRB nanoparticles, 57%, 73% and 47% of the active electrodes were bursting electrodes (> 1 burst / min and < 51 spikes per burst) per MEA on average at pretreatment, respectively. (**b**) Mean burst duration. (**c**) Mean number of spikes per burst was not systematically affected by MIRB dosages up to 10 µg/ml. (**d**) Mean burst rate. Error bars denote Poisson counting errors in (**a**) and the standard errors of the means in (**b**), (**c**), and (**d**). For the mean burst duration in (**b**), the p-values relative to the control (*p*_5_*, p*_10_), for 5 and 10 µg/ml respectively, were (0.004, 0.094), (0.994, 0.872), (0.540, 0.757), and (0.013, 0.997) for 0, 4, 24, and 48 h respectively. With the same notation, for the mean spikes per burst in (**c**), the *p*-values were (0.932, 0.022), (0.528, 0.982), (0.889, 0.955), and (0.994, 0.976). The *p*-values for the mean burst rate were (0.412, 0.175), (0.093, 0.987), (0.0003, 0.967), and (0.082, 0.770).

By visual inspection, healthy spiking and bursting activity was initially observed during pretreatment (0 h) on all MEAs prior to MIRB addition (Fig. 4). Among the active electrodes, some electrodes showed bursting while others showed monotonic spiking as expected from normal neuronal variation^44,45^. The MEA spiking and bursting electrophysiology parameter values reported in Fig. 5 and 6 are consistent with previously reported electrophysiological results for day in vitro (DIV) 19–21 murine neuron cultures^46,47^. In all dosage groups, the number of electrophysiologically active electrodes somewhat drops after MIRB or control medium application (Fig. 5a). The drop suggests that the physical disturbance arising from medium removal (four washes) may be responsible; mechanical fluid stress can reduce spiking in neuron sub-populations without causing significant cell death^48^.

For spike rate analysis, we found MEA neuron cultures incubated with 0 µg/ml (controls, *N* = 4, one control MEA per dose), 5 µg/ml (*N* = 3), and 10 µg/ml (*N* = 3) MIRB nanoparticles for 2 h performed similarly over the 48 h time course of observations (Fig. 5). In contrast, neuron cultures incubated with 20 µg/ml (*N* = 3) and 40 µg/ml (*N* = 2) showed clear toxicity, with a 95% decrease in active electrodes and a 30-fold drop in spike rate for the 20 µg/ml dose and a total cessation of spiking in the 40 µg/ml dose (Fig. 5). Because in the 40 µg/ml sample, all activity stopped at the 4 h time point onwards, we omitted this dose from further electrophysiology analysis. All active electrodes were averaged to calculate the mean spike rate of a given dose at each time point in the remaining samples.

We then performed more in-depth analysis on the subset of electrodes exhibiting bursting behavior. Burst analysis was not performed on MEA data at the 20 µg/ml or 40 µg/ml MIRB dosages, as those samples had zero bursting electrodes at the 4 h point. A small fraction of obvious outlier bursting electrodes with excessively large numbers of spikes per burst at a given time point and dose (> 51 spikes per burst, i.e., > 1 SD from the mean value), likely from a larger clump of neurons, were cut from the burst analysis after inspection of the data^49,50^. We verified by visual inspection that the MaxInterval method’s previously optimized standard burst identification parameters^51^ correctly identified bursts in our traces, with an example labeled bursting trace given in Supplementary Fig. 4.

All bursting electrodes were averaged to calculate the burst parameters of a given dose at each time point (Fig. 6a). Upon incubation with MIRB nanoparticles, using the Dunnett test, we found no statistically significant differences (p < 0.01) between the control and the 5 or 10 µg/ml dosage groups in either the mean burst duration (Fig. 6b) or the mean number of spikes in a burst (Fig. 6c). For the mean burst rate (Fig. 6d) there was no statistical difference between different doses and the controls, with the exception of a single anomaly in the 5 µg/ml dose that had a jump in burst rate at the 24 h time point. This anomaly could be spurious given that this behavior did not occur at other time points, or could be related to the burst rate parameter being highly sensitive to variations in local connectivity (which can change daily^46^) and in density of a neuron culture^46^.

Therefore, spike analysis of the MEA data showed that 20 and 40 µg/ml MIRB nanoparticle dosages (incubated for 2 h) significantly alter electrophysiological behavior of neuron cultures, while dosages of 10 µg/ml and lower do not cause statistically significant differences in either spiking or bursting behavior as compared to control cultures.

## Discussion

In this work, we have established clear toxicity thresholds for MIRB nanoparticles in murine neuron culture for both short (2 h) and longer (24 h) particle exposures, using both fluorescent (ROS and viability stains) and electrophysiological measures of neuronal viability. The lack of a dose-dependent increase in ROS (suggesting that the particle coating integrity is maintained) is qualitatively consistent with previous in vitro nanotoxicology studies in fibroblasts^19^ suggesting that there is a threshold concentration of internalized dIONPs that leads to cell death, rather than cell death arising from excess ROS generation^38^. To our knowledge, our report is the first in vitro nanotoxicology study of dIONPs with a strong positive surface charge using mature primary neuron cultures (DIV19-21).

Previous nanotoxicology studies of IONPs with various surface modifications and sizes for biomedical applications have reported various degrees of toxicity for both neurons^15,52,53^ and somatic cells^54–56^. Specifically, for neurons, dIONPs without amine functionalization and thus a near neutral surface charge, were tested by Rivet et al. on young (DIV2-3) chick neuron cultures. Using a combination of electron microscopy, Calcein AM, and propidium iodide, they tested cell viability and membrane integrity, ultimately finding no toxicity from the fluidMAG-D dIONP used in their study^52^. It is important to note that although they utilized propidium iodide, caution should be used when employing the dye in more mature neuron cultures where glial cells were not suppressed (such as those used in this work), as the connexin channels formed between glial cells and neuron cells can lead to significant uptake of propidium iodide even by healthy neuronal cells^57,58^.

By not inhibiting glial cells, which are part of the mammalian brain’s defense against iron toxicity^59^, we intended that the present study be directly relevant for in vivo comparison. Although changes in general mouse behavior were not observed with comparably sized dIONPs injected intraperitoneally into mice at a dose of 100 mg^60^, neuronal toxicity at the cellular level might still exist. Indeed, in another in vivo study in which embryonic and larval zebra-fish were exposed to comparably sized and coated dIONPs (with positive charged amine functionalization) in their aquatic environment, apoptotic cellular pathways were found to be activated, and corresponding behavioral changes were observed at dIONP dosages as low as 1 μg/ml^20^.

**Figure 7:**
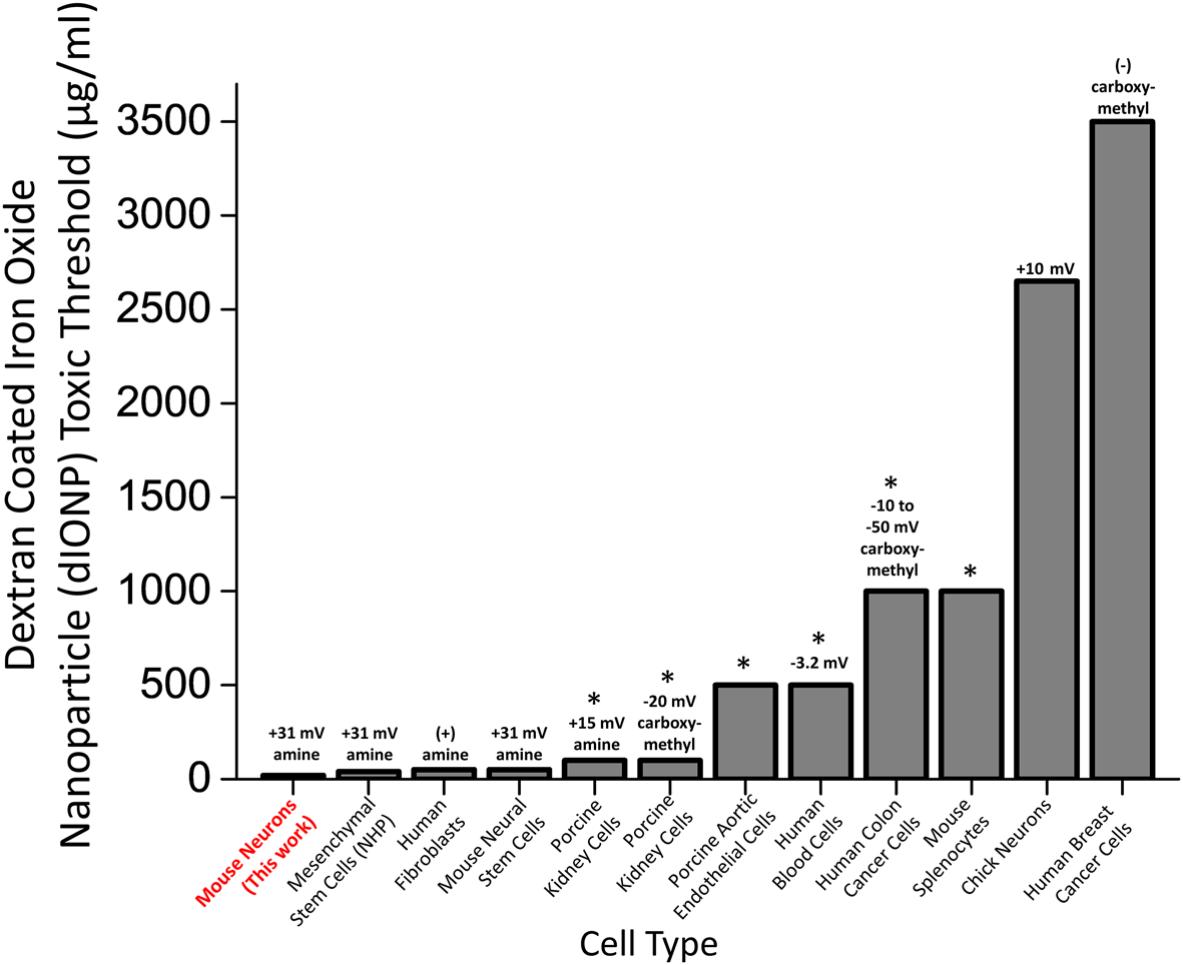
Dextran-coated iron oxide nanoparticle toxicity threshold for different cell types, zeta potentials, and functionalization groups. A comparison of threshold doses generating significant toxicity after exposure to ~ 10 nm-sized dextran coated iron oxide nanoparticle (dIONP). Each column lists the organism, tissue, particle functionalization and zeta potential: mouse neurons (this work) (amine functionalization), nonhuman primate mesenchymal stem cells^32^ (amine functionalization), human fibroblasts^61^ (amine functionalization), mouse neural stem cells^15^ (amine functionalization), porcine kidney cells^62^ (amine and carboxymethyl functionalization separately tested), porcine aortic endothelial cells^38^ (dextran only), human erythrocytes, monocytes, and leukocytes^26^ (dextran only), human colon cancer cells^63^ (carboxymethyl functionalization), mouse splenocytes^60^ (dextran only), primary chick neurons^52^ (dextran only), and human breast cancer cells^64^ (carboxymethyl functionalization), A (+) or (−) not accompanied by a numerical value indicates that the zeta potential was not reported in a given column’s study, but that we interpreted the amine and carboxymethyl functionalization as having a positive and negative charge respectively (as is standard). The absence of a zeta potential value indicates that the study used a dextran coating without functionalization, so that the sign of the surface charge is ambiguous. A “*” above a column indicates the highest dIONP dose value tested in that study, but that no gross toxicity effects were observed even at this dose. See each column’s reference for more information on the specific toxicity assays used for each study.

Our measured in vitro thresholds for lethal toxicity in primary neurons were lower than reported for other cell types exposed to identical (commercial MIRB nanoparticles) or similar (e.g. in-house prepared) ~ 10 nm iron core, amine functionalized dIONPs optimized for high efficiency cell uptake applications^15,32,61,62^ (Fig. 7). These results suggest that researchers should be cautious when extending toxicity results from more robust cell types (e.g. cancer cells or stem cells) to other somatic cells types. Furthermore, the toxicity threshold for primary neurons and other cell types exposed to amine-containing dIONPs is substantially lower than comparably sized dIONPs with either carboxymethyl functionalization^62–64^ (some of which also have strong cell uptake properties^62,63^) or native dextran hydroxyl functionalization (for lower cell uptake applications)^26,38,52,60^ (Fig. 7). Finally, it has been observed that coated IONPs with negative surface charges tend to accumulate within the liver and spleen within a few days, while positively charged coated IONPs aggregate most in the lungs^65^ (though this observation has not been verified for MIRB nanoparticles). The lungs are a less desirable location as the liver is one of the primary sites for iron metabolism and thus can likely break down excess particles more safely and efficiently^18^.

Our results demonstrate that MIRB nanoparticles, and possibly positively charged dIONPs generally, may be detrimental to primary neurons at significantly lower concentrations than previously reported for other cell types. The MIRB nanoparticles’ dextran coating with a positive charge (+ 31 mV zeta potential^32^) from amine functionalization of the core is standard in biocompatible ENP cell-uptake applications^13,15,32,37,41^, as increasing the positive surface charge is known to greatly enhance cell uptake efficiency^66–68^. However, higher uptake from greater positive surface charge may also be accompanied by an increased cytotoxicity^69–72^. In contrast, nanoparticles with dextran coating without amine groups leads to near neutral or moderately negative surface charge depending on the preparation method and particle solvent^26,62,63^. Although these nanoparticles are characterized by lower toxicity risks^38,52,70,73^, they also have a lower efficiency in cellular uptake and are thus likely more suitable for applications that do not require cell internalization (e.g. vascular imaging)^26^.

While it is tempting to attribute toxicity to positive surface charge from the amine groups, pinpointing the exact toxicity mechanism is challenging as it may result from multiple simultaneous risk factors that are difficult to resolve from one another, including particle charge, particle shape, particle size, coating and functionalization protocols, core nanomaterial selection, and cellular uptake rates (Fig. 7)^69–72^. Each particle type may also be internalized by multiple pathways that are difficult to untangle from each other, and which may also vary by cell type^72,74^. Now that the first wave of nanotoxicology research has clearly established the complexity of deconvoluting nanotoxicity factors and mechanisms arising from various nanoparticles (and for metal oxide nanoparticles especially^75^), recent studies have begun calling for a more systematic and comprehensive approach to nanotoxicology^39,75^.

In addition to encouraging caution in using MIRB nanoparticles, and IONPs in general, for CNS cellular uptake applications such as monitoring neuronal stem cell grafts^15^, cancer therapy^14^ and drug delivery^76^, our neurotoxicity results could contribute to predicting the effects of environmental exposure (e.g. occupational spills or release to the environment)^77^. As the potential for human exposure to dIONPs increases with increasing use of dIONPs in proposed and established medical procedures^13,73^, it is imperative that we fully understand the risks and impacts of these and related nanoparticles^75^.

## Methods

### Substrate preparation

Primary cultured neuron experiments were performed on two substrates: standard well plates and planar MEA culture chambers. Cell viability and ROS assays were performed in 24-well plates, whereas electrophysiology experiments used single-well MEAs with wells of comparable volume (~ 1 ml) to those in a 24-well plate. As is standard in neuron culture^78^, both substrates were coated with 0.1% polyethylenimine (PEI) (Sigma P3143) in borate buffer (Thermo Scientific 28341) followed by 10 μg/ml laminin (Sigma L2020) in cell medium, as laminin is a common supplement for better neuronal adhesion to MEAs^78^, and PEI is known to more effectively promote neuronal maturation than other adhesion layer chemicals (e.g. poly-D-lysine)^79^.

### Primary neuron culture

Brain tissue from the CNS of postnatal mice (Jackson Laboratory, C57BL/6, wild type) was harvested on postnatal day 0 or 1 following the standard BrainBits primary CNS neuron culture digestion protocol (https://www.brainbitsllc.com/primary-neuron-plating-protocol/), except with the papain digestion step lengthened from 10 to 30 min for postnatal tissue. Neural cells from digested brain tissue were densely plated at 500,000 cells per well to better mimic dense in vivo brain conditions. NbActiv4 was used as both the plating and feeding medium, as this serum-free medium is reported to be optimal for the electrical maturation of primary neurons while suppressing astrocyte growth^47^. Neuron cultures were incubated at 37 °C and 5% CO_2_ at all times other than during medium exchanges and MEA recordings. Neuron cultures had a medium exchange on the first day in vitro (DIV1), and then a medium exchange every 3–4 days. Microglial growth was not inhibited, as is preferred in electrophysiology studies^80^.

### MIRB addition to cultures

MIRB nanoparticles were readily internalized by cultured neurons (Fig. 1a, Supplementary Fig. 1) in our experiments. The critical physical properties of the MIRB nanoparticle include a zeta potential of + 31 mV in 1 mM KCl solution (very low aggregation), an effective diameter of 35 nm (optimal for cell uptake), and an iron core size of 8 nm with magnetic properties useful in MRI. These values have been verified in previous studies^15,32,37,81^. For MIRB manufacturer-provided measurements of these properties along with tunneling electron microscope (TEM) and confocal microscopy images of internalized MIRB nanoparticles (which were found to be stored entirely in cellular endosomes), see “Application Note 3” for Biopal product CL-50Q01-6A-50 (https://biopal.com/pdf-downloads/application-notes/application-note-3.pdf). Additional TEM and confocal microscopy images of internalized MIRB nanoparticles in neural stem cells and their derivative cells are also provided in previously published work^15,81^.

A medium exchange was always performed on DIV18, the day before MIRB addition. For neuron viability, ROS, and electrophysiology experiments alike, MIRB nanoparticles (2 mg/ml, original stock solution in dH_2_O) were added through a 20 μl droplet into each well. Wells were then stirred gently ~ 10 times until the media had a homogenous color change, to obtain the target concentration dose in each well (5, 10, 20, or 40 µg/ml) and for each incubation time (2, 24 or 48 h). DIV19 was chosen to ensure a sufficiently electrically mature culture^46^. A 20 μl drop of pure NbActiv4 medium was added to the control wells instead (one control MEA per dose test).

The shorter MIRB incubation times used herein (~ 2 h) are expected to better mimic the in vivo exposure time of particles. 2 h of dIONP exposure was chosen in previous in vitro nanotoxicology tests of fibroblasts^19^, and the manufacturer reports that MIRB nanoparticles have a blood half-life of several hours (https://biopal.com/mirb.htm)*.* However, longer incubation time data (24 and 48 h) was explored in well plates to provide time-dependent toxicity insight.

MIRB nanoparticles were imaged by a Texas-Red filter set on a Nikon TiE microscope after incubation for the target incubation time. Hoechst 33342 live cell nuclear staining was used to verify that the MIRB nanoparticles were co-localized with cells (Thermo Fisher R37605). The kinetics of MIRB association with neural cells was studied by incubating MIRB nanoparticles (at 5 and 20 µg/ml) in wells for 2 min, 15 min, 30 min, 45 min, or 60 min followed by fluorescent imaging under low magnification (10x). For this specific assay, each region of interest (ROI) was drawn around a small dense cluster of neurons to measure the mean fluorescent MIRB signal per ROI, and then the mean fluorescence of an adjacent region lacking any neurons was subtracted from the signal ROI to obtain the final fluorescence value.

### ROS assays and cell viability

To measure ROS levels in neurons exposed to different MIRB doses (5, 10, 20, and 50 µg/ml), a live cell Fluorometric Intracellular ROS Kit (Sigma MAK143) was used, following standard kit instructions. This kit is especially sensitive to superoxide and hydroxyl radicals and utilizes a proprietary fluorescent reporter^82^. Subsequent to MIRB incubation (2 or 24 h) but prior to imaging, ROS kit reagents were incubated with the neuron cultures for 1 h at 37 °C and 5% CO_2_. The MIRB nanoparticles were not washed out prior to the addition of the ROS reagents in order to rule out the possibility that cell culture changes resulted from mechanical disruption of the neurons during a buffer exchange. Fluorescent ROS imaging was performed on a Nikon TiE microscope using a FITC filter set.

To assess the intensity of ROS-induced fluorescence in individual neurons, which is indicative of their metabolic activity levels, we used ImageJ to draw tight regions of interest around neuron somas and measure fluorescence. Raw, unadjusted images without pixel saturation were used for the quantitative analysis. The average ROS level was determined for sets of 644, 464, 525, and 267 neurons at 0, 5, 10, or 20 µg/ml MIRB doses, respectively, at 2 h incubation times, and 403, 460, 81, and 24 neurons at 0, 5, 10, or 20 µg/ml doses, respectively, at 24 h incubation times. The accuracy of the threshold and cell identification was verified by inspection for each FOV. The average ROS level was determined for quasi-random subsets of representative neurons after background subtraction.

To count the number of metabolically active (i.e. live) neurons per field of view (FOV, ~ 3,500 μm^2^), ImageJ was used to count the number of fluorescent neuron somas per FOV over ~ 20 different regions. Quasi-random representative FOVs of neuron culture were chosen for doses with low or no toxicity, whereas for highly toxic doses (10 and 20 μg/ml at 24 h; 20 and 50 μg/ml at 48 h incubations), FOVs with the greatest densities of neurons in the entire well were chosen, thereby providing a conservative estimate of the MIRB toxicity. All well plate experiments were performed in duplicate.

### Calcein AM viability, metabolic, and membrane integrity assay

The viability and metabolism of the neuron cultures were further tested with an additional live cell fluorescent assay (Thermo Fisher A15001)^42^, after both 2 and 24 h incubation of the same doses used in the ROS assay. The “Cell Viability Indicator” (Calcein AM) component within this assay kit was used at 2 × concentration according to the standard kit instructions for 24-well plates. Calcein AM is also recognized as an indicator of membrane integrity^42,52^. The averaged mean fluorescence across representative FOVs for four and three replicate wells per dose were obtained for the 2 and 24 h incubation times respectively, and images across different doses were presented with equivalent exposure settings.

### Electrophysiology experiments

Electrophysiological recordings were made using single-well, 60-channel planar MEAs with 30 µm diameter titanium nitride electrode contacts in a 200 µm pitch, 8 × 8 array less corners (MEA2100-System, Multichannel Systems) (Fig. 1b, c).

On DIV19, MIRB nanoparticles were added to MEA cultures for 2 h and then washed out with fresh medium (four 50% washes), after which the cultures were returned to the incubator for stabilization until the time measurements were made. Cultures were removed from the incubator for MEA recordings at 4 (DIV19), 24 (DIV20), and 48 (DIV21) h post MIRB application.

MEA recordings were made by covering the wells with sealed lids (ALA Scientific ALAMEA-MEM5; Fig. 1b), which enable up to 30 min of stable CO_2_ levels in the culture medium when the MEA culture chambers are on the recording headstage outside an incubator^83^. MEA cultures (Fig. 1c) were maintained at 37 °C with a headstage hot plate during recording sessions. After at least 5 min of stabilization on the headstage^84^, recordings were performed for 5 min at a 10 kHz sampling rate^51,85^. MEA data were Nyquist low-pass filtered and high-pass filtered at 300 Hz for observation of fast action potentials^86^. Recordings, spike counting, and burst analyses were performed with the freely available Multichannel Systems Experimenter and Analyzer software packages (https://www.multichannelsystems.com).

Thresholds for spike identification were set to five standard deviations above noise (falling edge)^44^, and electrodes at a given dose and time point had to exhibit mean spike rates of > 0.03 Hz to be included in the spike rate analyses^44,84^. We called electrodes that met these criteria “active electrodes”. Electrodes in the burst analyses were required to have a threshold burst rate of > 1 burst per minute^87^ to be included; these we termed “bursting electrodes”. Spiking activity was scored for mean rate (spikes per second per electrode) and bursting properties, with individual bursts in each trace identified and characterized using previously optimized parameters for the MaxInterval method (included in the Multichannel Systems software) (Supplementary Fig. 4)^51^. All active electrodes and bursting electrodes at each dose and time point were pooled for their respective statistical analyses. Dramatic and obvious decreases in electrical activity occurred following addition of the lethal MIRB nanoparticles doses of 20 μg/ml and higher (Fig. 4; Fig. 5), while more detailed analysis was needed to investigate the electrical behavior at the sub-lethal doses (Fig. 6).

For both the spike rate and burst analyses, significant differences between doses at a given time point were tested by analysis of variance (ANOVA) followed by Dunnett’s multiple comparison post hoc test with a significance threshold of α = 0.01 and using 0 μg/ml as the common control^88^.

In all MEA experiments we refer to the pretreatment time point (~ 1 h before MIRB addition) as 0 h.

### Statement of ethical treatment of animals

The authors declare that institutional (Cornell University), state (New York), and federal (United States) guidelines and regulations were followed in the humane and ethical treatment of the animals used in this work. Our protocol (#2017-0079) was approved by Cornell University’s Institutional Animal Care and Use Committee.

## Supplementary information

Supplementary information

## Data Availability

This extended data repository is publicly available at 10.17605/OSF.IO/6XCKZ and includes the raw, unadjusted fluorescent images that were used in the ROS analysis and all raw MEA data used in this study, and the Multichannel Systems Analyzer software’s installation file and manual used to conduct the electrophysiology analysis in this report.
