# Supplementary material for: Dextran-coated iron oxide nanoparticle-induced nanotoxicity in neuron cultures
Source: Sci Rep. 2020 Jul 8;10:11239. doi: 10.1038/s41598-020-67724-w (PMC7343881; doi:10.1038/s41598-020-67724-w)
Supplement: Supplementary file 1 — Supplementary information [file 41598_2020_67724_MOESM1_ESM.pdf]

SUPPLEMENTARY INFORMATION

Dextran-Coated Iron Oxide Nanoparticle-Induced Nanotoxicity in Neuron Cultures

Ryan P. Badman[1], Shanna L. Moore[1,2], Jessica L. Killian[1,2], Tuancheng Feng[3,4], Thomas A. Cleland[5], Fenghua Hu[3,4], and Michelle D. Wang[1,2,*]

[1]Department of Physics & LASSP, [2]Howard Hughes Medical Institute, [3]Department of Molecular Biology and Genetics, [4]Weill Institute for Cell and Molecular Biology, [5]Department of Psychology, Cornell University, Ithaca, NY 14853, USA.
*Correspondence: mwang@physics.cornell.edu.

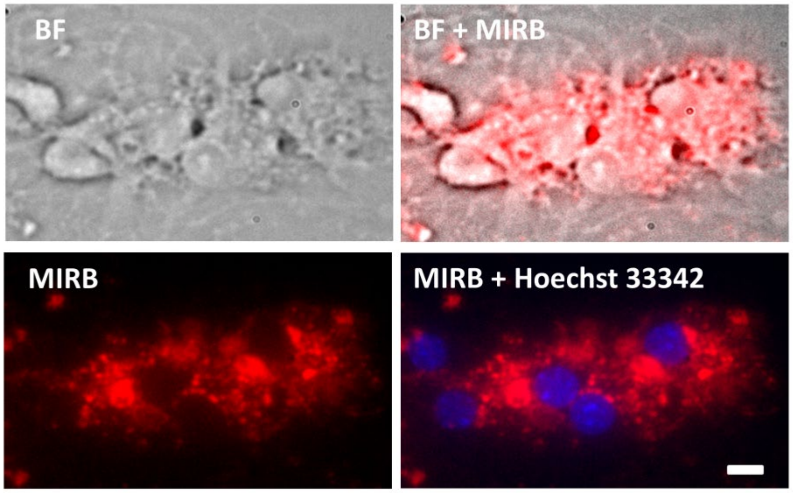

Supplementary Figure 1: MIRB Nanoparticle Uptake by Neurons

This shows another set of images, supplementary to those in the main text Figure 1a and with the same color scheme. DIV19 primary murine neurons plated on an MEA were incubated for 2 hr with 10 µg/ml MIRB nanoparticles before the particles were washed out leaving the neurons in fresh cell culture medium post MIRB-incubation. Both bright field and fluorescence images were acquired at 24 hr (DIV20) subsequent to the start of this incubation. The fluorescence image of MIRB particles is shown in red; the fluorescence image of Hoechst 33342 stain is shown in blue. Scale bars are 10 µm.

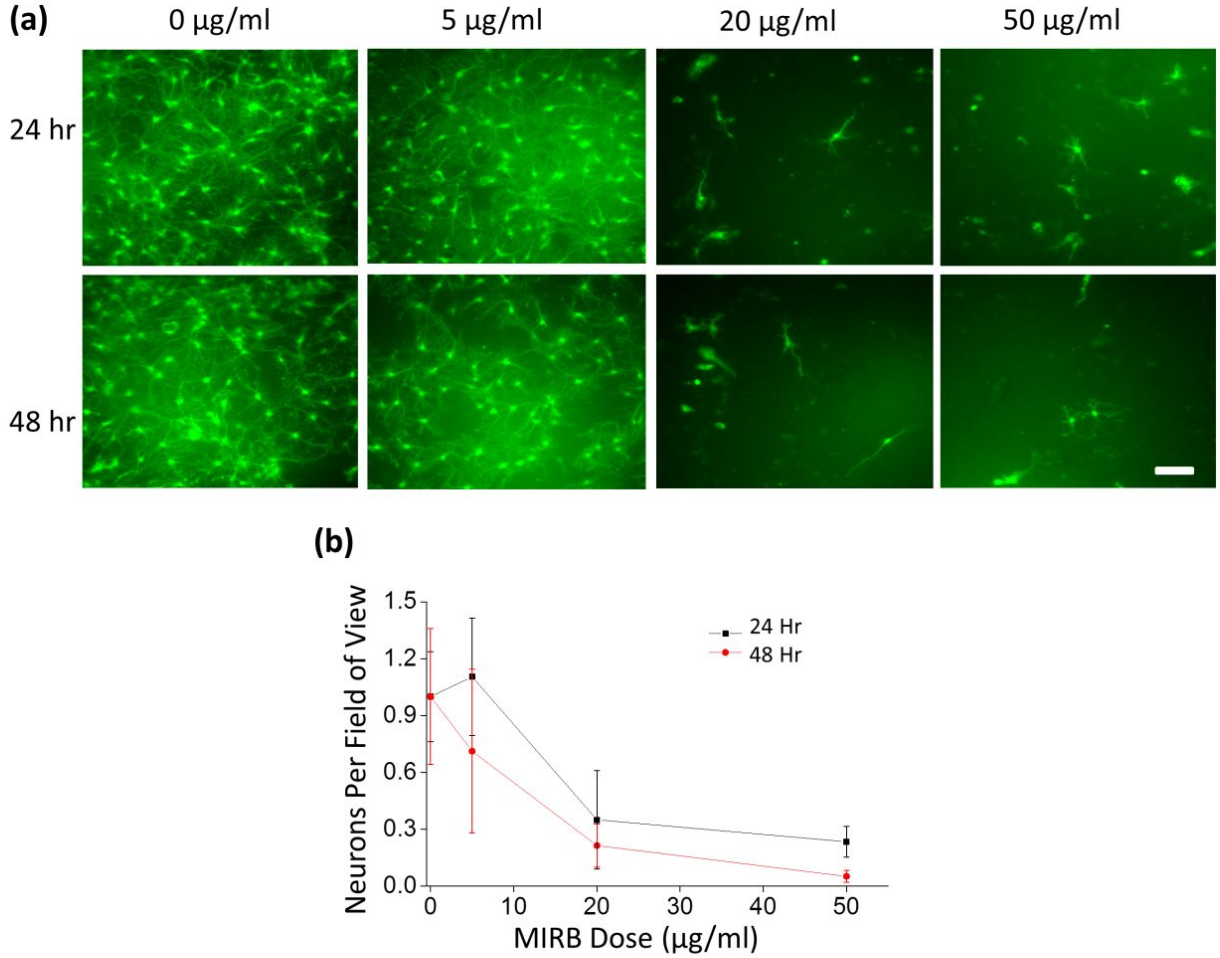

Supplementary Figure 2: ROS Staining of Neurons as a Viability Test

This figure is an extension to the main text Figure 3, obtained from a second experiment with different doses (0, 5, 20, 50 µg/ml) and incubation times (24 and 48 hr), but with otherwise identical methods as reported for Figure 3 (e.g. duplicate wells per dose). (a) DIV19 primary neurons were incubated with MIRB particles at one of four doses of MIRBs (0, 5, 20, or 50 µg/ml) for 24 or 48 hours followed by ROS staining. Live neurons were fluorescently observed with a proprietary fluorometric intracellular ROS kit (green) particularly sensitive to superoxide and hydroxyl radicals. Scale bar shown is 100 µm. (b) Plot of mean number of fluorescent neuron (somas) per field of view for the same image set as (a). Error bars are standard deviations.

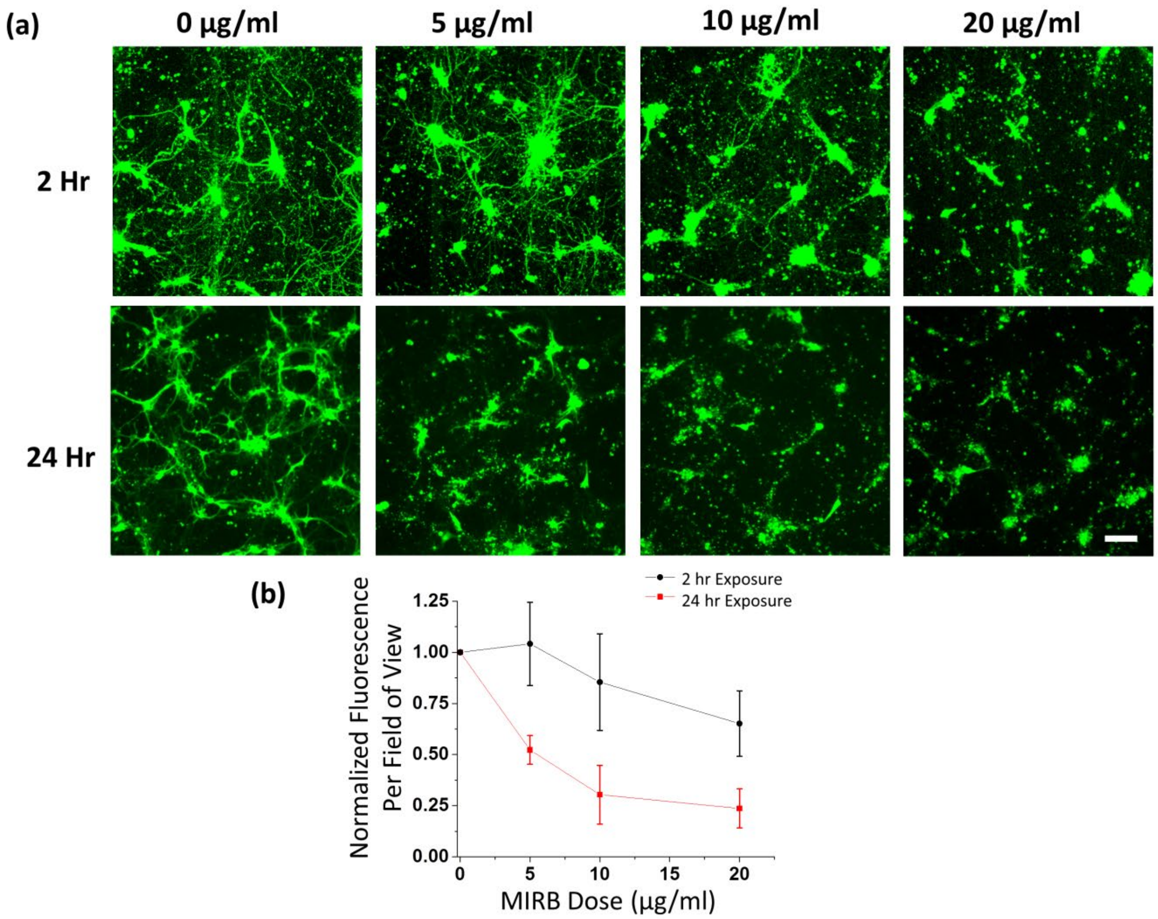

Supplementary Figure 3: An Additional Viability, Metabolic, and Cell Membrane Permeability Test of Neurons

(a) DIV19 neurons were incubated with MIRB nanoparticles for 2 hr (top row) or 24 hr (bottom row) at dosages of 0 µg/ml (control), 5 µg/ml, 10 µg/ml or 20 µg/ml. Live neurons were fluorescently observed with the cell membrane-permeable fluorescent reporter dye Calcein AM (green) that becomes fluorescent only upon interaction with enzymes in healthy, metabolically active cells. This dye is also recognized as an indicator of intact cell membranes. Scale bar is 100 µm. (b) Plot of the mean fluorescence intensity per FOV normalized against that of each replicate's control well (N = 4 for 2 hr, N = 3 for 24 hr). Each error bar denotes the standard deviation of the mean values of the replicate wells at a given time point and dose.

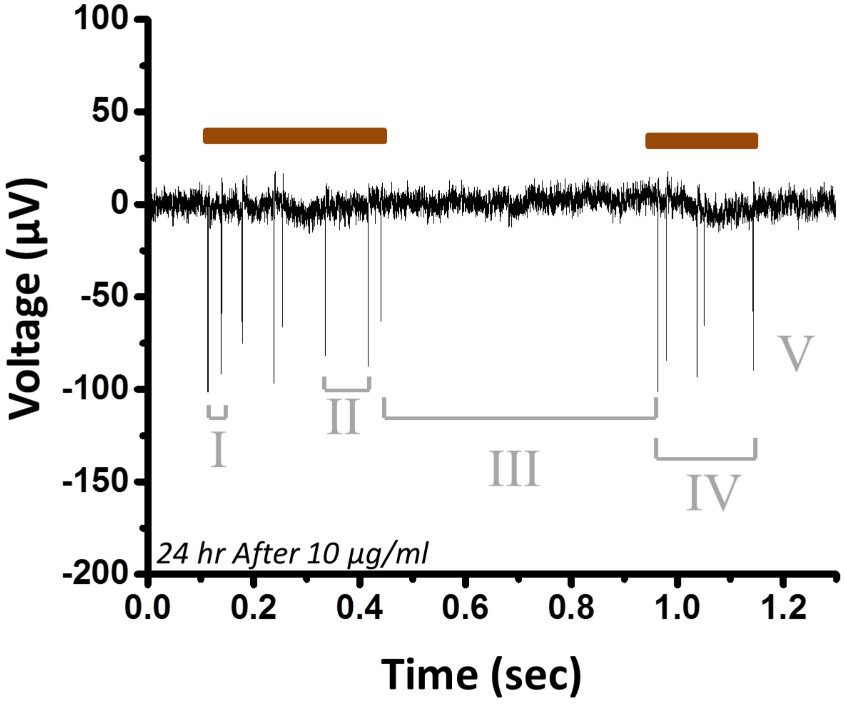

Supplementary Figure 4: Bursting Electrode Criteria

This shows an example electrophysiological recording (10 µg/ml dose, 24 hr time point) with bursts identified (brown horizontal line above bursts) using the following burst criteria in the MaxInterval method as labeled on the trace: (I) maximum interval of 170 ms between the first spikes in a burst, (II) maximum interspike interval of 300 ms allowed in a burst, (III) minimum interval of 200 ms between bursts, (IV) minimum burst duration of 10 ms, and (V) a minimum of 4 spikes per burst.
